# Common Variants rs429358 and rs7412 in *APOE* Gene Are Not Associated with POAG in a Saudi Cohort

**DOI:** 10.3390/biology13010062

**Published:** 2024-01-22

**Authors:** Altaf A. Kondkar, Tahira Sultan, Taif A. Azad, Tanvir Khatlani, Abdulaziz A. Alshehri, Essam A. Osman, Glenn P. Lobo, Faisal A. Almobarak, Saleh A. Al-Obeidan

**Affiliations:** 1 Department of Ophthalmology, College of Medicine, King Saud University, Riyadh 11411, Saudi Arabia; tasayed@ksu.edu.sa (T.S.); mtanwar@ksu.edu.sa (T.A.A.); eosman@ksu.edu.sa (E.A.O.); falmobarak@ksu.edu.sa (F.A.A.); salobeidan@ksu.edu.sa (S.A.A.-O.); 2 Glaucoma Research Chair in Ophthalmology, College of Medicine, King Saud University, Riyadh 11411, Saudi Arabia; 3 King Saud University Medical City, King Saud University, Riyadh 11411, Saudi Arabia; 4 Department of Blood and Cancer Research, King Abdullah International Medical Research Center, King Saud Bin Abdulaziz University of Health Sciences, Ministry of National Guard Health Affairs, Riyadh 11426, Saudi Arabia; khatlanita@mngha.med.sa; 5 Department of Ophthalmology, Imam Abdulrahman Alfaisal Hospital, Riyadh 14723, Saudi Arabia; abaalshehri@moh.gov.sa; 6 Department of Ophthalmology and Visual Neurosciences, University of Minnesota, Minneapolis, MN 55347, USA; lobo0023@umn.edu

**Keywords:** *APOE*, genetics, glaucoma, intraocular pressure, POAG, polymorphisms, rs429358, rs7412, Saudi

## Abstract

**Simple Summary:**

Glaucoma is a common eye condition linked to genes and aging. We studied genetic factors related to primary open-angle glaucoma (POAG) in adult Arabs from Saudi Arabia. We focused on two specific variations in apolipoprotein gene (*APOE*), namely rs429358 and rs7412, to examine whether these variations are more common in people with POAG. We compared the DNA from 179 people with POAG and 251 without. Our results showed that these genetic changes were not significantly linked to POAG. We also checked different combinations of these variations but did not observe a strong connection with POAG risk. The gene variations also did not affect eye pressure or other eye indicators, such as cup/disc, ratio linked to POAG. Overall, in our Saudi group, these specific gene variations do not seem to be major factors causing POAG. However, more studies with larger groups are needed to confirm this.

**Abstract:**

Adult-onset glaucoma, an age-related neurodegenerative disease, is very prevalent among the elderly Arabs of Saudi origin. This study investigated the association between apolipoprotein E (*APOE*) gene variants (rs429358 and rs7412) and primary open-angle glaucoma (POAG) in Arabs of Saudi origin. A case-control genetic association study involving 179 POAG patients and 251 controls utilized Sanger sequencing to genotype *APOE* gene variants. The allele frequencies and genotype distributions for rs429358 and rs7412 did not show significant associations with POAG. The haplotype analysis revealed apoε3 (87.6% and 87.4%) as the most prevalent, followed by ε4 (2.8% and 3.6%) and ε2 (9.6% and 8.9%) in the controls and POAG patients, respectively. Although the ε2/ε3 genotype and ε2-carriers displayed a more than two-fold increased risk, statistical significance was not reached. Notably, these polymorphisms did not affect clinical markers, such as intraocular pressure and cup/disc ratio. The logistic regression analysis demonstrated no significant influence of age, sex, rs429358, or rs7412 polymorphisms on POAG. In conclusion, within the Saudi cohort, *APOE* variants (rs429358 and rs7412) do not appear to be associated with POAG and are not substantial risk factors for its development. However, additional population-based studies are required to validate these findings.

## 1. Introduction

Adult-onset glaucoma is an age-related neurodegenerative disease and is very prevalent among the elderly Arabs of Saudi origin [1]. Primary open-angle glaucoma (POAG), a common form of glaucoma, is a complex, multifactorial ocular disorder characterized by elevated intraocular pressure (IOP), progressive retinal ganglion cell (RGC) death, optic nerve damage, and visual field loss [2]. It represents a significant global health concern, with an estimated 79.6 million individuals projected to be affected by 2020, a number expected to increase to 111.8 million by 2040 [3]. While the precise etiology of POAG remains elusive, genetic factors have been demonstrated to play a pivotal role in its pathogenesis, with numerous genome-wide and candidate-gene association studies highlighting the importance of genetic predisposition in disease susceptibility in various ethnicities [4,5]. Nonetheless, the underlying contribution of genes and genetic polymorphisms in such complex polygenic disease in POAG patients of Saudi Arabian descent is still unclear.

Apolipoprotein E (*APOE*) is a well-studied gene located on chromosome 19q13.32, known for its critical involvement in lipid metabolism and transport [6]. It exists in three major isoforms, denoted as ε2, ε3, and ε4, which are the result of two common single-nucleotide polymorphisms (SNPs): rs429358 (Cys112Arg) and rs7412 (Arg158Cys) [6]. These polymorphisms give rise to six possible genotypes, each contributing to variations in APOE protein function and expression levels [7]. Emerging evidence suggests that *APOE* polymorphisms may be implicated in the development and progression of various neurodegenerative diseases, including Alzheimer’s disease (AD), Parkinson’s disease, and multiple sclerosis [8,9]. However, the potential association between *APOE* polymorphisms and POAG has garnered increasing interest in recent years, as both conditions share common underlying pathological mechanisms, including oxidative stress, inflammation, and vascular dysfunction [10,11,12,13].

The APOE protein is expressed in the retina and optic nerve head tissues [14]. The ε4/ε4 genotype has been reported to be associated with increased risk of POAG in Asians [15,16,17], but not in populations from the United Kingdom [18] or Sweden [19]. According to certain recent reports, E4 has a protective effect on the development of POAG [20] or a favorable correlation with glaucoma risk [21]. However, others have discovered a conflicting relationship or a negative correlation between *APOE* E4 and glaucoma [22]. Therefore, although studies have suggested a potential link between *APOE* variants and POAG, the evidence is conflicting, with varying degrees of outcomes in different population [23,24]. 

The Saudi population presents a unique genetic landscape, characterized by distinct allele frequencies and genetic variations, which may contribute to the variability in disease susceptibility and presentation. Despite this, limited studies have investigated the role of *APOE* variations in Saudi patients with POAG [25]. However, more research is needed to clarify the role of this gene in POAG susceptibility in this population. Therefore, the present study aims to examine the association between the rs429358 and rs7412 variants of *APOE* and POAG in a Saudi cohort.

## 2. Materials and Methods

### 2.1. Ethical Considerations

This retrospective case–control study was conducted in accordance with the principles outlined in the Declaration of Helsinki. Ethical approval was obtained from the Institutional Review Board/Ethics Committee prior to the commencement of the study (protocol number #09–657). Informed written consent was obtained from all participants after providing them with a detailed explanation of the study objectives, procedures, and potential risks involved.

### 2.2. Study Participants

A total of 179 patients clinically diagnosed with POAG and 251 age- and ethnically-matched controls were recruited for this study. All participants were of Saudi Arabian descent and provided informed consent prior to inclusion. The diagnosis of POAG was based on established clinical criteria, including characteristic optic-nerve-head changes, visual field defects, and elevated IOP. The IOP assessment employed Goldmann applanation tonometry, while the cup-to-disc ratio was ascertained through the comparison of the vertical diameter of the optic cup, representing the central depression in the optic nerve head, with the vertical diameter of the entire optic disc. Exclusion criteria for both cases and controls encompassed the presence of secondary glaucomas, congenital anomalies, previous ocular surgeries, and other significant ocular or systemic comorbidities [26].

### 2.3. DNA Preparation

Genomic DNA was extracted from peripheral EDTA blood samples using the QIAamp DNA Mini Kit protocol, as suggested by the manufacturer (cat. no. 51306, Qiagen, Hilden, Germany). The DNA aliquots were stored at –80 °C until further use.

### 2.4. Genotyping of APOE Variants rs429358 (T>C) and rs7412 (C>T) 

The rs429358 (T>C) and rs7412 (C>T) polymorphisms of the *APOE* gene were genotyped using Sanger sequencing. The region encompassing the polymorphisms were PCR-amplified using primers and conditions described in Table 1. The PCR reaction consisted of standard reagents: 1X PCR buffer, 250 µM dNTP mix, 100 pmoles of each specific primer, 1.5 U Taq polymerase, and 20 ng of DNA, along with 1X Q-solution (cat. no. 203205, Qiagen). The PCR products were purified using a QIAquick PCR Purification Kit (cat. no. 28106, Qiagen) and subjected to sequencing using M13 primers in both forward and reverse directions using the BigDye Terminator V3.1 Cycle Sequencing kit (Applied Biosystems, Foster City, CA, USA), according to the manufacturer’s protocol. Samples were electrophoresed on the ABI 3730 XL sequencer (Applied Biosystems). The sequencing data were read using CLC Sequence Viewer 6.0 (Qiagen, Hilden, Germany) to identify the nucleotide variations and *APOE* genotypes in comparison to *APOE* reference sequence (NG_007084.2).

### 2.5. Statistical Analysis

Demographic data, including age and sex distribution, were compared between cases and controls using Mann–Whitney U test (for continuous variables) and chi-squared tests (for categorical variables). Deviations from Hardy–Weinberg equilibrium for *APOE* genotypes in control subjects were assessed using chi-squared tests.

The association between *APOE* variants (rs429358 and rs7412) and POAG risk was evaluated using logistic regression models, adjusting for potential confounding variables, such as age and sex. Odds ratios (ORs) and 95% confidence intervals (CIs) were calculated. The potential association of polymorphisms with clinical factors such as IOP and cup/disc ratio was determined by non-parametric method in dominant model. Furthermore, a binary logistic regression model was applied to assess the combined effect of age, sex, rs429358, and rs7412 on the likelihood of developing POAG.

All statistical analyses were performed using SPSS version 25 (IBM Inc., Chicago, IL, USA) and SNPStats web tool (https://www.snpstats.net/start.htm (accessed on 11 December 2023)), and *p*-values < 0.05 were considered statistically significant. Power analysis was performed using the PS program (version 3.1.2).

## 3. Results

### 3.1. Demographic Characteristics

**Figure 1:**
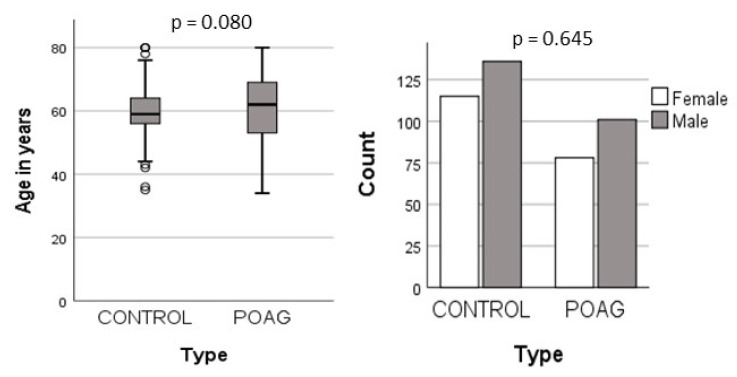
Demographic distribution in POAG and controls.

In this study, a total of 430 samples were analyzed, comprising 179 controls and 251 POAG patients. The mean ages of the two groups were 61.1 (±10.1) years for the POAG patients and 59.7 (±7.0) years for the controls, with no statistically significant difference in age. Furthermore, there were 101 (56%) males and 78 (44%) females in the patient group, compared to 136 (54%) males and 115 (46%) females among the controls. The gender distribution also showed no significant variations between the two groups (Figure 1).

### 3.2. Allele and Genotype Associations

The minor allele frequencies (MAFs) for rs429358 and rs7412 were determined in both the controls and the POAG patients. Both polymorphisms were found to be in Hardy–Weinberg equilibrium. The MAFs for rs429358 were 0.10 and 0.09 in the controls and the POAGs, respectively, while for rs7412, they were 0.03 and 0.04 in the controls and the POAGs, respectively. No significant associations were observed between the allele frequencies and the occurrence of POAG (Table 2).

The genotype associations were assessed under different genetic models, including co-dominant, dominant, and recessive models. However, none of these models revealed a significant association between the *APOE* polymorphisms and the POAG patients. Although the frequency of the rs7412 C/T heterozygous genotype was higher in the POAGs (7.3%) than in the controls (4.8%), exhibiting a 1.55-fold increased risk of POAG. However, this difference did not reach statistical significance (Table 3).

### 3.3. Association of APOE Genotypes with POAG

**Figure 2:**
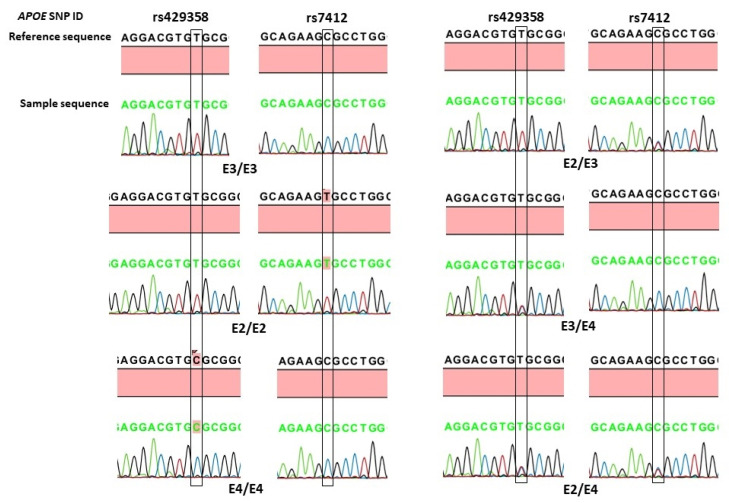
Representative DNA-sequence chromatograms of *APOE* genotypes based on rs429358 (T>C) and rs7412 (C>T) variants. The position of the nucleotide change is boxed.

In this study, we also examined the association of the two *APOE* variants according to the three *APOE* alleles (ε3, ε2, and ε4) and six different genotypes (ε3/ε3, ε2/ε2, ε2/ε3, ε2/ε4, ε3/ε4, and ε4/ε4). The *APOE* allele and genotype calling was based on the presence of the nucleotide sequences shown in Table 4, and their association analysis is shown in Table 5. The *APOE* ε3 was the most prevalent allele, followed by ε4 and ε2, in both the POAG patients and the controls. However, no significant associations were observed between the *APOE* alleles and the occurrence of POAG. The most common genotype in both the cases and the controls was ε3/ε3, and the overall distribution of different *APOE* genotypes did not show statistical significance (Pearson chi-squared = 4.61, df = 5, *p* = 0.465). The homozygous ε2/ε2 and ε4/ε4 genotypes were absent in the POAGs. The frequency of the heterozygous ε2/ε3 genotype was 1.6% in the controls, compared to 4.5% in the POAGs, showing a 2.8-fold increased risk of POAG. A further analysis of the *APOE* genotypes according to carrier status (Table 3) showed that the frequency of ε2 carriers (ε2/ε2 and ε2/ε3 genotypes) was also high in the POAGs (4.6%), compared to the 2.0% in the controls, exhibiting more than two-fold increased risk of POAG. However, none of these differences reached statistical significance (Table 3). The representative chromatograms of *APOE* sequencing of the rs429358 (T>C) and rs7412 (C>T) variants and the identified *APOE* genotypes is shown in Figure 2.

### 3.4. Regression Analysis

The effects of risk factors including age, gender, and *APOE* gene variants on POAG risk were assessed through binary logistic regression analysis. The results demonstrated no significant impact of age (*p* = 0.098), sex (*p* = 0.622), rs429358 (*p* = 0.939), or rs7412 (*p* = 0.605) on the risk of developing POAG. The effect of the variants was examined in both co-dominant and dominant models (Table 6).

### 3.5. Association with Clinical Variables

**Figure 3:**
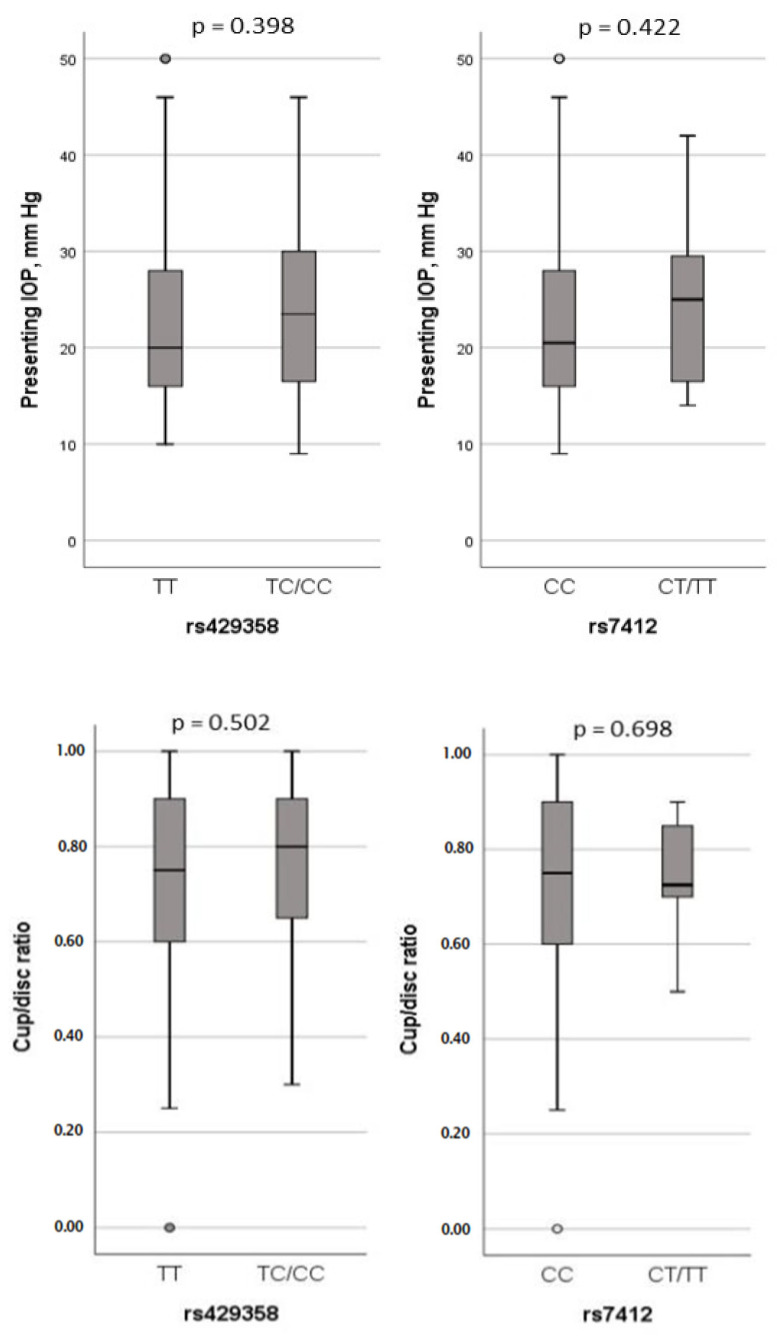
Association analysis of IOP and cup/disc ratio with rs429358 and rs7412 genotypes in dominant model.

The study further examined the potential association between *APOE* polymorphisms and clinical parameters of POAG, including IOP and cup/disc ratio. However, no significant effects of either SNP on these clinical variables were observed (Figure 3).

## 4. Discussion

In our study, we explored the potential association between two prevalent polymorphisms of the *APOE* gene, rs429358 and rs7412, and POAG. We conducted an analysis to assess the independent association of these polymorphisms and their haplotypes in relation to *APOE* alleles/genotypes.

Specifically, for the rs429358 polymorphism, where T is the reference allele and C is the alternate allele, we observed C allele frequencies of 0.1 in the controls and 0.09 in the POAG cases within our cohort. This frequency was higher than in Europeans (0.07) and Asians (0.037), but lower than in Africans (0.13) and African Americans (0.13), according to NCBI ALFA allele frequency data. Notably, our study did not reveal any allelic or genotype associations of rs429358 polymorphism with POAG. Additionally, this polymorphism did not have an impact on IOP or cup/disc ratio.

For the rs7412 polymorphism, where C is the reference allele and T is the alternate allele, the T allele frequency was 0.03 in the controls and 0.04 in the POAG cases. Compared to the ALFA allele frequency data, this frequency was higher than in Latin Americans (0.026) and South Asians (0.021), but much lower than in Europeans (0.083), Africans (0.10), African Americans (0.10), and East Asians (0.07). As with rs429358, this polymorphism did not demonstrate a significant effect on POAG or related clinical factors, such as IOP and cup/disc ratio.

Regarding the haplotypes of these polymorphisms (rs429358 and rs7412), referred to as *APOE* alleles and genotypes, numerous studies have been conducted. In most populations, ε3 is reported as the most common allele, followed by ε4 and ε2 [27]. Our study aligned with this pattern, with the prevalence of *APOE* alleles found to be in the following order of ε3 > ε4 > ε2. Nevertheless, our study did not detect any significant associations between *APOE* alleles/genotypes and POAG. Furthermore, the examination of the association of *APOE* genotypes with POAG also yielded no significant findings. It is worth noting that the ε2/ε3 genotype appeared to increase the risk of POAG by over two-fold, although this effect did not reach statistical significance. This trend was consistently observed when considering ε2-carrier status. Similarly, no significant impact of *APOE* ε2, ε3, or ε4-containing genotypes on IOP and cup/disc ratio was observed in the dominant models. Moreover, the binary logistic regression analysis showed no significant influence of age, sex, rs429358, or rs7412 polymorphisms on POAG outcomes. In summary, our study demonstrated no significant associations between *APOE* alleles/genotypes and POAG, or its associated clinical markers, such as IOP and cup/disc ratio, within our Saudi cohort.

The APOE protein is a prominent apolipoprotein predominantly expressed in the central nervous system, synthesized by retinal Müller cells, neurons, and macrophages [14,28]. It plays a pivotal role in neural growth and repair processes. Associations between Apo E4 and neurodegenerative disorders, such as AD, and eye-related disorders, like AMD, have been documented [9,29]. Furthermore, POAG, characterized by a significant hereditary component, is also considered a neurodegenerative disorder. Since early reports of the *APOE* ε4 allele’s association with increased risk of normal tension glaucoma in the Tasmanian population [30], several investigators have examined this link in adult-onset POAG. However, the results regarding the association between *APOE* alleles/genotypes and POAG have been contradictory.

In a German study, it was noted that the presence of the ε2 allele influenced IOP levels in normal controls [31]. In Japanese patients with open-angle glaucoma (OAG), the ε3 allele was associated with an increased risk of OAG, while the ε2 allele was linked to a significant reduction in OAG risk, and the ε4 allele was associated with lower IOP levels [32]. In contrast to our findings, a smaller study conducted by Al-Dabbagh et al. in Riyadh, which included 60 Saudi-origin POAG patients, reported a significant association between the ε4 allele and POAG [25]. A large-scale study utilizing data from the NEIGHBOR Massachusetts Eye and Ear Infirmary dataset reported a protective effect of the ε4 allele in both high-tension and normal-tension POAG patients in this population [20]. A protective effect of the ε4 allele was also observed in the Canadian Longitudinal Study on Aging, in which it was found to be protective against glaucoma in individuals without systemic hypertension [21]. Conversely, in Brazilian POAG patients, individuals carrying the ε2 allele were reported to be at an increased risk of developing POAG [33]. In contrast, Mullany et al. reported that the ε4 allele was associated with an increased rate of neuroretinal thinning in eyes with normal-tension glaucoma [22]. Our study did not replicate the findings of these studies.

While numerous studies have emphasized the involvement of *APOE* in POAG, a subset of investigations has yielded contrasting results regarding the genetic association between *APOE* and POAG across diverse ethnic groups. Our own study is in agreement with these divergent findings. Notable examples include studies conducted on European [18,19,34], Chinese [15,35], Japanese [36], and Turkish [23] populations, as well as other meta-analysis studies [24,37]. The distribution of *APOE* allele frequencies, as observed in these distinct studies and our current research, is summarized in Table 7. Notably, the frequencies of the ε2, ε3, and ε4 alleles exhibit variability among different ethnicities. Across all populations, ε3 consistently emerges as the prevailing allele, followed by ε4 and ε2, in descending order of prevalence.

Several factors may contribute to the disparities in *APOE* findings in POAG. These encompass the potential impact of clinical diversity within the study population, variations in functional properties of different APOE isoforms in different cell types [7], and potential interactions with other genetic elements involved in lipid metabolism or neuroinflammation [28,38]. It is also crucial to recognize that genetic associations are inherently complex and can diverge across populations due to age, sex, race, and differing genetic backgrounds and environmental influences [39]. Moreover, discrepancies in sample size or study power can also contribute to conflicting results, even within the same population, as reported by Mullany et al. in the UK population [34], by Al-dabbagh et al. in patients of Saudi origin [25], and our study. In our study, considering the observed allele frequency, the estimated power to detect an odds ratio of 2.0 at a significance level of 0.05 was 0.6 per allele for ε2 and 0.9 per allele for ε4. However, to substantiate our findings, a significantly larger sample size would be necessary, especially when aiming to detect an odds ratio of 1.5, which is commonly observed in genetic association studies with more subtle effects. 

POAG is a complex polygenic and multifactorial disease. Given the strong evidence for the role of *APOE* in AD [9], the role of *APOE* in glaucoma is still unclear and the exact mechanisms by which *APOE* might contribute to the risk of the development and progression of glaucoma or may be protective needs further investigation. Although our study reports no associations between the two common variants, rs429358 and rs7412, and POAG, the role of other polymorphism(s), including the promoter polymorphism, which influences APOE expression, and those in linkage disequilibrium, cannot be ruled out and needs to be investigated. 

## 5. Conclusions

Our findings indicate that there is no apparent link between these *APOE* variants and POAG within our cohort of individuals of Saudi Arabian descent. This implies that *APOE* might not be a significant risk factor for POAG in this particular ethnic group. However, further population-based studies are required to validate these results.

## Figures and Tables

**Table 1:** PCR primers and cycling condition used for *APOE* genotyping.

PCR Primers	Primer Sequences (5′–3′)	Amplification Condition
Forward	TGTAAAACGACGGCCAGTGACCATGAGGAGTTGAAGGCCTAC	95 °C for 15 min Cycling: 35 cycles
Reverse	CAGGAAACAGCTATGACCGATGGCGCTGAGGCCGCGCT	95 °C—1 min 59 °C—30 s 72 °C—1 min
		3. 72 °C—10 min

Underlined sequences represent M13 sequences that were used for sequencing.

**Table 2:** Minor allele frequency distribution of *APOE* polymorphisms.

SNP ID	Gene/ Locus	Chromosome	Position *	Minor Allele	MAF		OR (95% CI)	*p*
					Controls	POAG		
rs429358	*APOE*	19q13.32	44908683	C	0.10	0.09	1.05 (0.57–1.91)	0.887
rs7412	*APOE*	19q13.32	44908821	T	0.03	0.04	1.14 (0.40–3.25)	0.806

* Genomic base-pair position as per GRCh38/hg38. MAF—minor allele frequency, OR—odds ratio, 95% CI—95% confidence interval.

**Table 3:** Genotype analysis of rs429358 and rs7412 variants in *APOE* gene in POAG.

SNP/Model	Genotype	Control	POAG	OR (95% CI)	*p* ^§^	AIC	BIC
rs429358							
Codominant	T/T	204 (81.3)	147 (82.1)	1.00	0.580	588.9	601.1
	T/C	46 (18.3)	32 (17.9)	0.97 (0.59–1.59)			
	C/C	1 (0.4)	0 (0)	0.00 (0.00–NA)			
Dominant	T/T	204 (81.3)	147 (82.1)	1.00	0.820	587.9	596.1
	T/C-C/C	47 (18.7)	32 (17.9)	0.94 (0.57–1.55)			
Recessive	T/T-T/C	250 (99.6)	179 (100)	1.00	0.300	586.9	595
	C/C	1 (0.4)	0 (0)	0.00 (0.00–NA)			
	T/C	46 (18.3)	32 (17.9)	0.97 (0.59–1.60)			
rs7412							
Codominant	C/C	238 (94.8)	166 (92.7)	1.00	0.330	587.8	600
	C/T	12 (4.8)	13 (7.3)	1.55 (0.69–3.49)			
	T/T	1 (0.4)	0 (0)	0.00 (0.00–NA)			
Dominant	C/C	238 (94.8)	166 (92.7)	1.00	0.370	587.2	595.3
	C/T-T/T	13 (5.2)	13 (7.3)	1.43 (0.65–3.17)			
Recessive	C/C-C/T	250 (99.6)	179 (100)	1.00	0.300	586.9	595.0
	T/T	1 (0.4)	0 (0)	0.00 (0.00–NA)			

§ *p*-value was not significant for age and sex adjustment; AIC—Akaike information criterion, BIC—Bayesian information criterion; NA—indicates outside measureable range.

**Table 4:** *APOE* allele and genotype calling based on the presence of nucleotide sequences at rs429358 and rs7412 variants.

*APOE* Variants	rs429358	rs7412
Alleles		
ε2	T	T
ε3	T	C
ε4	C	C
Genotypes		
ε3/ε3	TT	CC
ε2/ε2	TT	TT
ε4/ε4	CC	CC
ε2/ε3	TT	TC
ε3/ε4	TC	CC
ε2/ε4	TC	TC

**Table 5:** Association analysis of *APOE* variants according to *APOE* alleles and genotypes in POAG.

APOE	Controls *n* (%)	POAG *n* (%)	Odds Ratio (95% Confidence Interval)	*p*
Alleles				
ε3	440 (87.6)	313 (87.4)	1.00	-
ε2	14 (2.8)	13 (3.6)	1.30 (0.60–2.81)	0.497
ε4	48 (9.6)	32 (8.9)	0.89 (0.28–2.79)	0.841
Genotypes				
ε3/ε3	199 (79.3)	139 (77.6)	1.00	-
ε2/ε2	1 (0.4)	0 (0)	0.00 (0.00–NA)	0.999
ε2/ε3	4 (1.6)	8 (4.5)	2.80 (0.84–9.69)	0.078
ε2/ε4	8 (3.2)	5 (2.8)	0.89 (0.28–2.79)	0.841
ε3/ε4	38 (15.1)	27 (15.1)	1.01 (0.59–1.74)	0.999
ε4/ε4	1 (0.4)	0 (0)	0.00 (0.00–NA)	0.999
Carrier ^a^				
ε3/ε3	199 (81.9)	139 (79.9)	1.00	-
ε*2 ^b^	5 (2.0)	8 (4.6)	2.29 (0.73–7.150)	0.143
ε*4 ^c^	39 (16.0)	27 (15.5)	0.99 (0.58–1.70)	0.999

^a^ ε2/ε4 were excluded from either ε*2 or ε*4 group. ^b^ includes ε2/ε2 and ε2/ε3. ^c^ includes ε4/ε4 and ε3/ε4. Overall Pearson chi-squared = 4.612, df = 5, *p* = 0.465. NA—indicates outside measureable range.

**Table 6:** Binary logistic regression analysis.

Group Variables	B	SE	Wald	Odds Ratio	95% Confidence Interval	*p*
Age	0.019	0.12	2.73	1.02	0.99–1.044	0.098
Sex	0.098	0.198	0.243	1.10	0.74–1.62	0.622
rs429358			0.126	-	-	0.939
T/C	−0.094	0.263	0.126	0.911	0.543–1.526	0.723
C/C	−21.236	40192.970	0.000	0.000	-	1.000
T/C + C/C	−0.096	0.261	0.134	0.910	0.54–1.51	0.714
rs7412			1.006	-	-	0.605
C/T	0.429	0.428	1.006	1.536	0.664–3.55	0.316
C/C	−21.070	40192.970	0.000	0.000	-	1.000
C/T + C/C	0.355	0.418	0.720	1.42	0.62–3.23	0.396

**Table 7:** *APOE* allele frequencies reported in POAG patients with different ethnicities.

Ethnicity	Cases *n*	Controls *n*	Cases Frequency %			Controls Frequency %			Reference
			ε2	ε3	ε4	ε2	ε3	ε4	
German	96	32	12	71	17	14	62	24	[31]
Japanese	310	179	2.6	91.4	6.0	5.0	84.0	10.6	[32]
Saudi Arabs	60	130	0	90.5	9.5	0	95.7	4.2	[25]
Canadian	1093	23,562	4.1	78.1	17.8	2.6	77.9	19.5	[21]
Brazilian	402	401	8.6	79.9	11.4	6.11	81.8	12.1	[33]
Australian	1161	2571	7.7	77.4	14.9	8.4	77.1	14.5	[22]
European	137	75	12.8	72.6	14.6	10.7	76.0	13.3	[18]
Chinese	400	281	10.7	82.7	6.6	8.5	82.2	9.4	[15]
Japanese	28	77	5.4	75.0	19.6	7.8	87.7	4.5	[36]
Swedish	484	374	10.3	77.7	12.0	11.2	77.3	11.5	[19]
Chinese	176	200	10.3	9.7	79.5	9.0	8.7	82.2	[35]
Turkish	75	119	8.7	84.0	7.3	4.2	85.7	10.1	[23]
European	13,988	56,894	8.0	77.0	15.0	8.0	76.8	15.1	[34]
Saudi Arabs	179	251	3.6	87.4	8.9	2.8	87.6	9.6	This study

## Data Availability

The data supporting the conclusions of this article are all presented within the report.
